# Prevalence of microcephaly and Zika virus infection in a pregnancy cohort in Kenya, 2017–2019

**DOI:** 10.1186/s12916-022-02498-8

**Published:** 2022-09-14

**Authors:** Eric Osoro, Irene Inwani, Cyrus Mugo, Elizabeth Hunsperger, Jennifer R. Verani, Victor Omballa, Dalton Wamalwa, Chulwoo Rhee, Ruth Nduati, John Kinuthia, Hafsa Jin, Lydia Okutoyi, Dufton Mwaengo, Brian Maugo, Nancy A. Otieno, Harriet Mirieri, Mufida Shabibi, Peninah Munyua, M. Kariuki Njenga, Marc-Alain Widdowson

**Affiliations:** 1 Washington State University Global Health Kenya, One Padmore Place, George Padmore Road, Off Ngong Road, Nairobi, Kenya; 2 Paul G. Allen School of Global Health, Washington State University, Pullman, USA; 3 Department of Pediatrics and Child Health/Kenyatta National Hospital, University of Nairobi, Nairobi, Kenya; 4 Division of Global Health Protection, Centers for Disease Control and Prevention, CDC Kenya, Nairobi, Kenya; 5 Center for Global Health Research, Kenya Medical Research Institute, Nairobi, Kenya; 6 Division of Global Health Protection, CentersforDiseaseControlandPrevention, Atlanta, USA; 7 Research and Programs Department, Kenyatta National Hospital/University of Nairobi, Nairobi, Kenya; 8 Coast General Hospital, Mombasa, Kenya; 9 Department of Obstetrics and Gynecology/Kenyatta National Hospital, University of Nairobi, Nairobi, Kenya; 10 Institute of Tropical and Infectious Diseases, University of Nairobi, Nairobi, Kenya; 11 Port Reitz Hospital, Mombasa, Kenya; 12 Institute of Tropical Medicine, Antwerp, Belgium

**Keywords:** Zika virus, Microcephaly, Pregnancy, Kenya

## Abstract

**Background:**

Zika virus (ZIKV), first discovered in Uganda in 1947, re-emerged globally in 2013 and was later associated with microcephaly and other birth defects. We determined the incidence of ZIKV infection and its association with adverse pregnancy and fetal outcomes in a pregnancy cohort in Kenya.

**Methods:**

From October 2017 to July 2019, we recruited and followed up women aged ≥ 15 years and ≤ 28 weeks pregnant in three hospitals in coastal Mombasa. Monthly follow-up included risk factor questions and a blood sample collected for ZIKV serology. We collected anthropometric measures (including head circumference), cord blood, venous blood from newborns, and any evidence of birth defects. Microcephaly was defined as a head circumference (HC) < 2 standard deviations (SD) for sex and gestational age. Severe microcephaly was defined as HC < 3 SD for sex and age. We tested sera for anti-ZIKV IgM antibodies using capture enzyme-linked immunosorbent assay (ELISA) and confirmed positives using the plaque reduction neutralization test (PRNT_90_) for ZIKV and for dengue (DENV) on the samples that were ZIKV neutralizing antibody positive. We collected blood and urine from participants reporting fever or rash for ZIKV testing.

**Results:**

Of 2889 pregnant women screened for eligibility, 2312 (80%) were enrolled. Of 1916 recorded deliveries, 1816 (94.6%) were live births and 100 (5.2%) were either stillbirths or spontaneous abortions (< 22 weeks of gestation). Among 1236 newborns with complete anthropometric measures, 11 (0.9%) had microcephaly and 3 (0.2%) had severe microcephaly. A total of 166 (7.2%) participants were positive for anti-ZIKV IgM, 136 of whom became seropositive during follow-up. Among the 166 anti-ZIKV IgM positive, 3 and 18 participants were further seropositive for ZIKV and DENV neutralizing antibodies, respectively. Of these 3 and 18 pregnant women, one and 13 (72.2%) seroconverted with antibodies to ZIKV and DENV, respectively. All 308 samples (serum and urine samples collected during sick visits and samples that were anti-ZIKV IgM positive) tested by RT-PCR were negative for ZIKV. No adverse pregnancy or neonatal outcomes were reported among the three participants with confirmed ZIKV exposure. Among newborns from pregnant women with DENV exposure, four (22.2%) were small for gestational age and one (5.6%) had microcephaly.

**Conclusions:**

The prevalence of severe microcephaly among newborns in coastal Kenya was high relative to published estimates from facility-based studies in Europe and Latin America, but little evidence of ZIKV transmission. There is a need for improved surveillance for microcephaly and other congenital malformations in Kenya.

**Supplementary Information:**

The online version contains supplementary material available at 10.1186/s12916-022-02498-8.

## Background

In 2015, the Asian lineage of Zika virus (ZIKV), a mosquito-borne pathogen first described in Uganda in 1947, spread globally to cause widespread outbreaks in the Americas and Asia. Although more than 80% of these ZIKV infections were mild or subclinical, infections in pregnant women were associated with multiple congenital fetal malformations collectively referred to as congenital Zika syndrome and include microcephaly, congenital contractures, brainstem dysfunction, and severe cerebral and eye lesions [1–4].

In February 2016, the World Health Organization declared the ZIKV outbreak a public health event of international concern, and calls were made for pregnancy cohort studies to better understand the role of clinical and subclinical infections in congenital malformations and characterize the incidence and spectrum of these adverse outcomes [5]. Subsequently, prospective studies in Latin America during the outbreak reported a wide range of seropositivity to ZIKV infection (8–53%) and confirmed the association between Zika infection in pregnant women and adverse fetal outcomes [1, 6, 7].

In sub-Saharan Africa (sSA), two outbreaks of ZIKV (Asian lineage) associated with microcephaly were reported in Cape Verde and Angola in 2016 [8, 9]. Although modeling studies suggest that the risk of transmission of flaviviruses, including ZIKV, dengue, and yellow fever, is high in many areas of sSA [10], limited data exist on ZIKV circulation in this region. The surveillance of microcephaly in the continent is very rudimentary, and it remains unclear if ZIKV infection has caused an undocumented burden of microcephaly and other birth defects [11]. One recent analysis of specimens from a dengue outbreak has shown that ZIKV co-circulated in Kenya in 2013, while another study found a prevalence of neutralizing anti-ZIKV antibodies of up to 7% in Northern Kenya [12, 13]. However, cross-reacting antibodies between flaviviruses can complicate the interpretation of these findings [14]. Moreover, serologic and viral surveillance cannot easily differentiate between the Asian and African lineages of Zika. Recent studies suggest that the African ZIKV lineage virus has higher transmissibility and pathogenicity compared to the Asian lineage strain, and infection in pregnant women may be more likely to cause total fetal loss than congenital deformities associated with the Asian lineage [15].

We established a prospective cohort study among pregnant women in an area of coastal Kenya predicted to be at risk for circulating ZIKV [16] because of circulating dengue and the *Aedes aegypti* vector, the primary mosquito that transmits ZIKV. We aimed to determine the incidence and seroprevalence of ZIKV infection and assess its association with adverse pregnancy outcomes.

## Methods

### Study setting

We enrolled pregnant women who sought care at one of three hospitals in Mombasa on the east coast of Kenya. These were (a) Coast General Hospital, the second largest public hospital in Kenya with a capacity of 700 beds; (b) Port Reitz Hospital with a capacity of 166 beds; and (c) Bomu Hospital, a private health hospital with a capacity of 45 beds, and an HIV care reference center.

### Participants and eligibility criteria

Pregnant women presenting for antenatal care (ANC) were eligible for enrolment if they met the following criteria: (1) aged 15 years or older, (2) had a confirmed pregnancy from ANC records or ultrasound, (3) had an estimated gestational age ≤ 28 weeks, and (4) planned to attend ANC and deliver at the study hospitals. We excluded women who were found to have an ectopic or molar pregnancy by ultrasound findings and were participating in trials of experimental drugs and devices or those incarcerated.

### Study procedures

After informed consent and enrolment, a baseline questionnaire was administered, a dating ultrasound was conducted for those without an obstetric ultrasound before enrolment, and 5 ml of venous blood sample was collected. When ultrasound was not available, the date of the last menstrual period, antenatal medical records on gestational age, or clinical assessment of fundal height were used in that order to assess gestational age. Participants were followed up monthly at the study clinic for the duration of their pregnancy at which time a questionnaire on risk factors was completed and a 5-ml blood sample collected. Participants were also asked to report and attend the clinic for assessment if they experienced any fever or rash. If symptom onset was within 7 days of the report, urine and blood samples were collected.

**Fig. 1:**
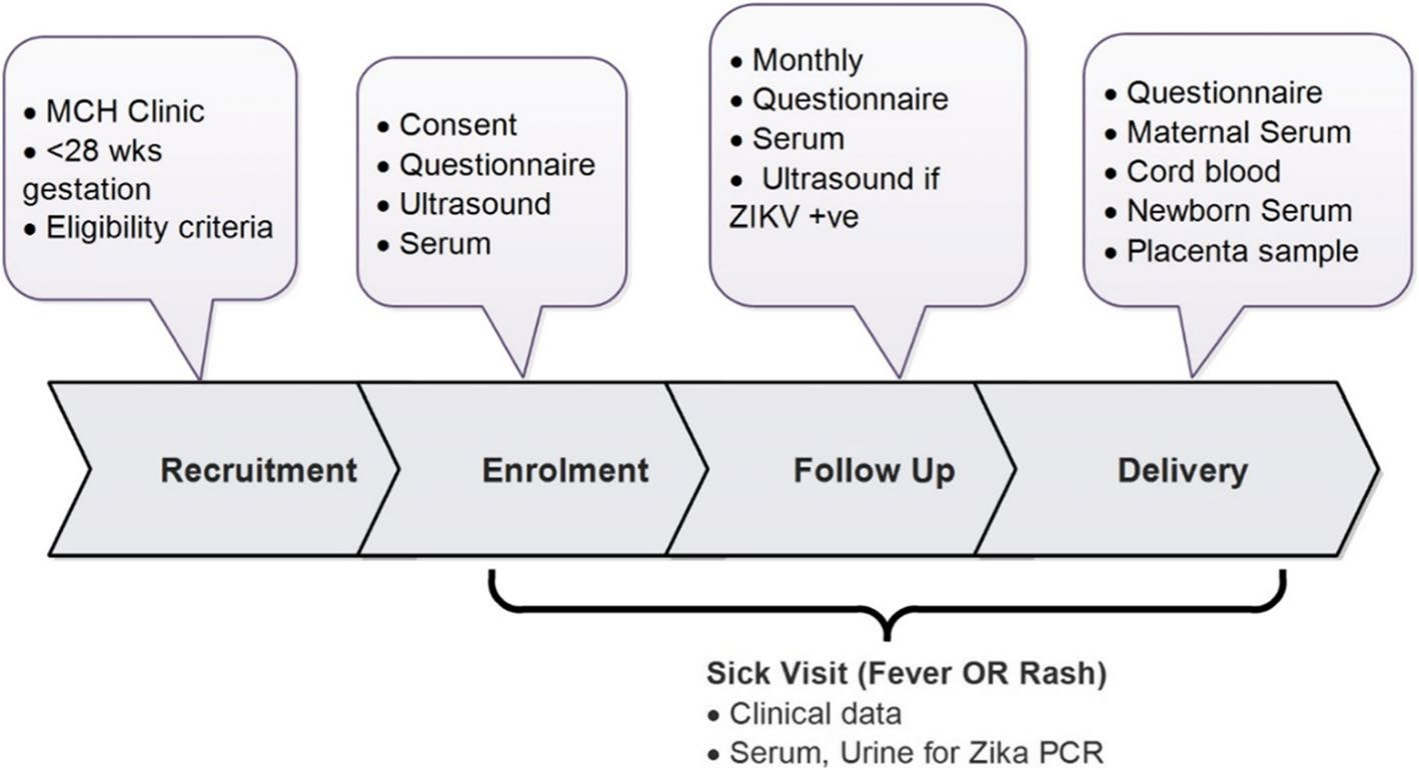
Schedule of study procedures in the pregnancy cohort, Kenya, 2017–2019

At delivery, maternal, placenta, and cord blood samples were collected, and a questionnaire administered. The study staff conducted a newborn physical assessment within 48 h of birth for gross abnormalities and measured birth weight, head circumference, and other anthropometric characteristics. Additionally, 3 ml of venous blood was drawn from the newborn (Fig. 1). Participants who did not deliver at the study sites were asked to attend the study facilities within 2 weeks for maternal and newborn blood sample collection.

### Data collection

During enrolment and monthly follow-up, structured questionnaires were administered by trained study staff to collect data on sociodemographic characteristics, pregnancy characteristics, potential risk factors for congenital defects, and environmental risk factors for ZIKV infection such as water storage, exposure to mosquitoes, and prevention against mosquito bites. Other data collected included medical and obstetric history, signs and symptoms of possible ZIKV infection, and other relevant infections, including details and timing in relation to pregnancy/gestational age and pregnancy outcomes (such as live birth, miscarriage, and stillbirth). Study data were collected via electronic tablets using REDCap [17]. We abstracted data from participants’ ANC, sick visits, and delivery medical records to capture relevant information. Clinical measurements of the newborn were performed following standard procedures by study staff at the study sites [18].

### Laboratory procedures

All samples were barcoded, stored at − 20 °C, and shipped to the Kenya Medical Research Institute/Centers for Disease Control and Prevention laboratory in Nairobi for storage at − 80 °C until tested in the same laboratory.

**Fig. 2:**
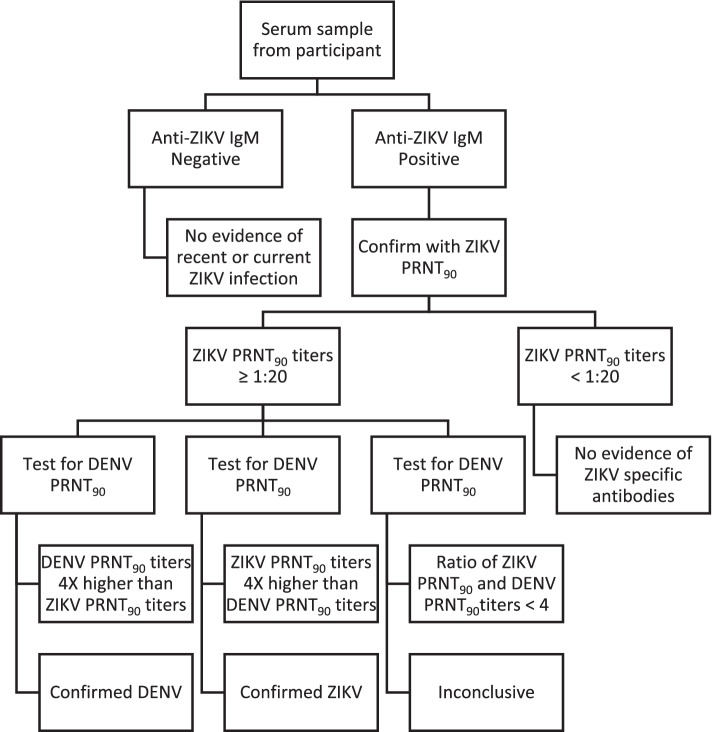
Testing algorithm for Zika virus antibodies among participants in the pregnancy cohort, Kenya, 2017–2019. DENV, dengue fever virus; ZIKV, Zika virus; PRNT, plaque reduction neutralization assay

Maternal and newborn sera were tested for anti-ZIKV IgM antibodies using the ZIKV IgM antibody capture enzyme-linked immunoassay (MAC-ELISA) as described previously [19]. Briefly, Immunol plates were coated with anti-human IgM (Kirkegaard and Perry Laboratories) and incubated overnight before incubation for 2 h with patient sera diluted at 1:400. Vero-cell-derived ZIKV E6 antigen was added to the plate and detected using 6B6C1 anti-ZIKV IgG horseradish peroxidase-conjugated monoclonal. Due to the potential of cross-reactivity with other flaviviruses (particularly dengue virus), anti-ZIKV IgM-positive samples were confirmed using the ZIKV 90% plaque reduction neutralization test (PRNT_90_) as previously described [20, 21]. Furthermore, samples with ZIKV PRNT_90_ titers ≥ 1:20 were tested for dengue virus-2 (DENV2, used because it cross-reacts with other DENV serotypes) by PRNT_90_ (Fig. 2). Urine and serum samples from participants with possible ZIKV infection (defined below) and anti-ZIKV IgM positive were tested for ZIKV RNA using CDC real-time reverse transcription polymerase chain reaction (rRT-PCR).

### Study definitions

A possible case of ZIKV infection was defined as any participant with fever (≥ 38° C) or a history of fever (within the previous 7 days) or a rash. A probable case was defined as the presence of IgM antibodies against ZIKV. A confirmed case of ZIKV infection was defined as detection of ZIKV RNA by rRT-PCR in serum or urine samples, or ZIKV IgM positive and PRNT_90_ for ZIKV with titer ≥ 20 and ZIKV PRNT_90_ titer ratio ≥ fourfold higher when compared to DENV [22]. A confirmed DENV-positive case was defined as DENV PRNT_90_ titer ratio ≥ fourfold higher when compared to ZIKV PRNT_90_ titers (Fig. 2).

Microcephaly was defined as a head circumference < 2 standard deviations (SD) below the mean for sex and gestational age and severe microcephaly as a head circumference < 3 SD based upon INTERGROWTH-21st standards [18]. We defined stillbirth as a baby born without any sign of life and birthweight of ≥ 500 g or, if missing, ≥ 22 completed weeks of gestation. Abortion was defined as pregnancy losses < 22 weeks of gestation. Low birth weight was defined as < 2500 g. Small-for-gestational-age (SGA) was defined as a birth weight *z*-scores of <  − 1.28 at birth (equivalent to the 10th percentile) and extreme SGA as a birth weight of <  − 1.88 *z*-scores (equivalent to the 3rd percentile) [18].

### Sample size

The sample size estimation was based on the hypothesis that pregnant women with incident ZIKV infection (primary exposure) have a higher risk of microcephaly and other congenital malformations (primary outcome) in their offspring at delivery. Assuming a background risk of microcephaly of 0.03%, a minimum detectable relative risk of 25, and the proportion of the study cohort with incident Zika virus infection of 3 to 5% [23], a sample size of between 2127 and 2669 pregnant women was needed to achieve a power of at least 80%.

### Data analysis

Descriptive analyses were performed using categorical and continuous variables, compared using the chi-square test or Fisher’s exact test or using the Student *t*-test. Continuous variables with a non-normal distribution were compared using the Kruskal–Wallis test. For all the analyses, data were considered significant at a *p*-value of < 0.05. All data were analyzed using the R statistical software [24].

### Ethical considerations

This study was approved by the Kenyatta National Hospital/University of Nairobi Ethical Review Committee [P71/102/2017], the Washington State University institutional review board [IRB No. 15897], and the CDC institutional review board [# 7021]. Participants (those below 18 years were considered emancipated minors) provided informed consent at the time of recruitment.

## Results

### Characteristics at enrolment and follow-up

**Fig. 3:**
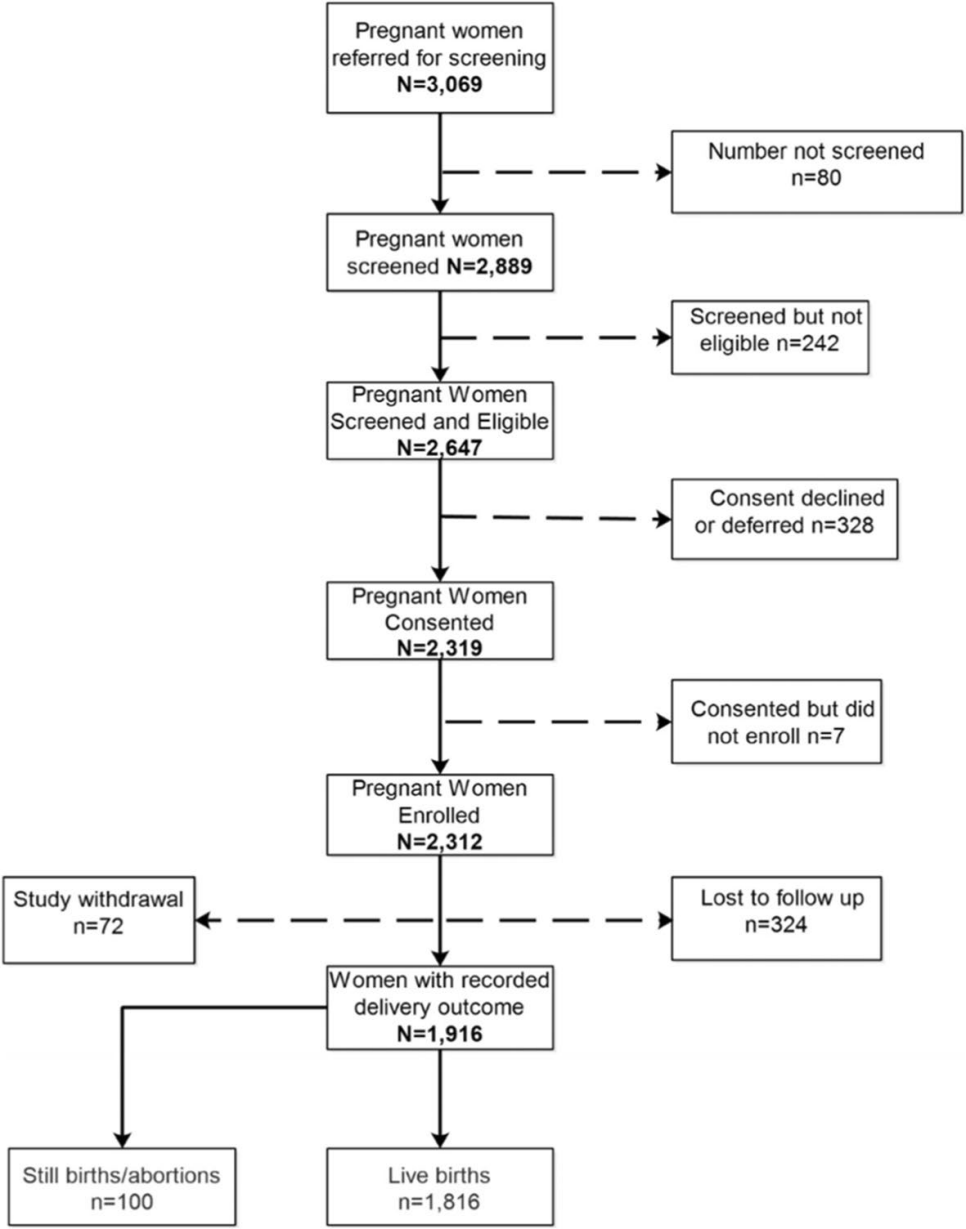
Flow diagram of the pregnancy cohort, Kenya, 2017–2019

From October 2017 to July 2019, a total of 3069 pregnant women were referred from the ANC clinics and 2889 (94.1%) were screened for eligibility. Of these, 2647 (91.6%) were eligible and 2312 (87.3%) enrolled in the study. Among enrollees, delivery outcome data were available for 1916 (82.9%) while 396 (17.1%) were either lost to follow-up (delivery outcome not recorded) or withdrew from the study (Fig. 3). Participants who were lost to follow-up or withdrew from the study were significantly older and with higher gestational age at enrolment (Table S1).

The mean age of the enrolled participants was 28.4 years (SD 5.6), median gestational age of 20.0 weeks [interquartile range (IQR) 15.1–24.1] at enrollment. A total of 2028 (87.5%) participants had a dating ultrasound completed. Overall, 343 (15.9%) of the participants were HIV infected, and of these, 267 (77.8%) were enrolled at Bomu hospital, and 310 (92%) were on antiretroviral medication. Nearly 30% of the participants reported a previous diagnosis of chikungunya or dengue by a health care worker. Other sociodemographic and obstetric characteristics are included in Table 1. The median number of follow-up visits among participants was four (IQR = 2–5). One hundred and four women reported fever (*n* = 99) or rash (*n* = 10) during pregnancy with 20 identified at enrolment and 84 during follow-up. Of all the symptomatic pregnant women, blood and urine samples were obtained from 81 (77.9%).

**Table 1:** Sociodemographic, obstetric, and clinical characteristics of the pregnancy cohort at enrolment, Kenya, 2017–2019

**Characteristic**	***N***	
Age, years mean (SD)	2312	28.4 (± 5.6)
Education level completed	2278	
Primary and below, *n* (%)		592 (26.0)
Secondary, *n* (%)		1016 (44.6)
Tertiary, *n* (%)		670 (29.4)
Occupation	2312	
Employed, *n* (%)		953 (41.2)
Self-employed, *n* (%)		366 (15.8)
Unemployed, *n* (%)		992 (42.9)
Gestational age in weeks, median [IQR]	2312	20.0 [15.1–24.1]
Previous pregnancies	2309	
0, *n* (%)		691 (29.9)
1 to 3, *n* (%)		1415 (61.3)
Above 3, *n* (%)		203 (8.8)
Previous pregnancies’ outcome	1618	
Pregnancy loss		528 (32.6)
Newborn with birth defects		19 (1.2)
Any chronic disease^a^, *n* (%)	2312	143 (6.2)
Systolic blood pressure (mmHg)^b^, mean (SD)	2309	109.3 (11.7)
Diastolic blood pressure (mmHg)^c^, mean (SD)	2307	67.9 (9.3)
Hemoglobin level (g/dl)^d^, mean (SD)	2145	11.2 (2.7)
HIV infected, *n* (%)	2160	343 (15.9)
HIV infected and using ARVs, *n* (%)	343	310 (92.3)
Positive VDRL for syphilis, *n* (%)	2163	29 (1.3)

*IQR* interquartile range, *SD* standard deviation, *VDRL* Venereal Disease Research Laboratory, *ARV* antiretroviral drugs

^a^Chronic disease included asthma, hypertension, diabetes, and epilepsy

^b^Normal range 90–120

^c^Normal range 60–80

^d^Normal range 9.5–15

### Delivery outcomes

Of 1916 recorded delivery outcomes, 1816 (94.8%) were live births (including 25 (1.3%) twin gestations) and 100 (5.2%) deliveries were either stillbirths or abortions (Table 2). More than two-thirds [1365 (71.2%)] of the deliveries were at the study facilities. A total of 23 (1.2%) of all newborns had at least one overt congenital abnormality: 12 (52.2%) congenital umbilical hernias, 6 (20.7%) upper and 4 (13.8%) lower limb deformities, and one Down syndrome. One newborn had both upper and lower limb deformities. Among the 66 stillbirths, 19 (28.7%) were examined at delivery with no gross anomalies noted.

**Table 2:** Delivery and newborn characteristics of the pregnancy cohort, Kenya, 2017–2019

Characteristic	Total	*n* (%)
Delivery outcome	1916	
Live newborn		1816 (94.8)
Stillbirth		66 (3.4)
Abortion (< 22 weeks)		34 (1.8)
Birth weight categories	1432	
< 2500 g		83 (5.8)
2500–4000 g		1272 (88.8)
> 4000 g		77 (5.4)
Newborn anthropometric measures		
Microcephaly (head circumference < 2SD)	1236	11 (0.9)
Severe microcephaly (head circumference < 3SD)	1236	3 (0.2)
Small for gestational age (birth weight < 1.8SD)	1200	142 (11.8)
Extremely small for gestational age (birth weight < 2.8SD)	1200	57 (4.8)

*SD* standard deviation

Among 1236 (64.5%) newborns with data on sex and gestational age and head circumference measurements, 11 (0.9%) presented with microcephaly and 3 (0.2%) with severe microcephaly. This translates to a prevalence of 90 (95% CI 45–159) cases of microcephaly per 10,000 births and 20 (95% CI 5–71) cases per 10,000 births for severe microcephaly. Slightly over 10% of the newborns were small for gestational age (SGA) (Supporting information).

### Laboratory results

We collected and tested a median of 4 (IQR 2–5) serum samples per participant, for a total of 8276 samples including 6518 (78.8%) maternal, 924 (11.2%) newborn, and 834 (10.1%) cord blood samples. During the study, 81 women presented with fever or rash. All serum and urine samples collected within 7 days of onset were negative by RT-PCR for ZIKV and by MAC-ELISA for IgM.

Samples from 2293 (99.2%) were tested for anti-ZIKV IgM, and 166 (7.2%) women had at least one blood sample positive for anti-ZIKV IgM. Among them, 131 (78.9%) tested positive in one sample, 25 (15.1%) in two samples, and 10 (6%) in more than two samples for a total of 213 positive IgM samples. The majority (136 [81.9%]) of these participants were anti-ZIKV IgM negative at enrolment and became seropositive during the follow-up period corresponding to an anti-ZIKV IgM incidence of 5.9 per 100 pregnancies. Two seropositive participants reported a history of clinical diagnosis of dengue fever within 4 weeks before collection of the sample that was anti-ZIKV IgM positive.

The proportion of anti-ZIKV IgM-seropositive participants who had newborns with microcephaly compared to seronegative participants was not statistically different (1.6% vs 0.8%, *p*-value = 0.682, *n* = 1236). Similarly, the proportion of SGA among anti-ZIKV IgM seropositive compared to seronegative participants was not statistically different (15.4% vs 11.4%, *p*-value = 0.245, *n* = 1200) (Table 3). There was no statistical association between ZIKV IgM seroconversion and microcephaly or SGA.

**Table 3:** Comparison of pregnancy and newborn characteristics by anti-Zika virus IgM positivity in the pregnancy cohort, Kenya, 2017–2019

Characteristic	Total	Anti-ZIKV IgM		*p*-value
		**Positive**	**Negative**	
Age in years at enrolment, median [IQR]	2293	26.7 [23.6–31.6]	28.1 [24.2–32.4]	0.158
Gestational age in weeks at enrolment, median [IQR]	2293	19.5 (14.0–23.0)	20.0 (15.1–23.9)	0.153
Small-for-gestational-age newborns, *n* (%)	1200	19 (15.4)	123 (11.4)	0.245
Microcephaly in newborns, *n* (%)	1236	2 (1.6)	9 (0.8)	0.682

*IQR* interquartile range, *ZIKV* Zika virus

Among the 166 participants with anti-ZIKV IgM, samples from 144 (86.7%) participants were tested by PRNT and 3 (2.1%) were confirmed ZIKV positive and 18 (12.5%) confirmed DENV positive while 7 (4.9%) were inconclusive, for a seroprevalence of 0.1% for ZIKV in the cohort (*n* = 2293). None of the newborns from the three ZIKV PRNT-positive women had microcephaly. Of the three participants positive for ZIKV by PRNT, two were positive at enrolment. The pregnancy and delivery characteristics of the three participants are outlined in Table 4.

**Table 4:** Pregnancy and delivery characteristics of three participants of a pregnancy cohort with confirmed ZIKV by PRNT, Kenya, 2017–2019

Characteristic	Participant 1	Participant 2	Participant 3
No. of samples positive/tested by ELISA for anti-ZIKV IgM	3/5	2/6	3/6
No. of samples positive/tested by PRNT	1/3	2/2	1/3
IgM positive at enrolment	Yes	Yes	No
Gestational age at PRNT confirmation	• Enrolment at 12.3 weeks	• Enrolment at 13.9 weeks • Follow-up at 24.8 weeks	• Follow-up at 33.3 weeks
Gestational age at enrolment	12.3 weeks	13.9 weeks	14.3 weeks
Age in years at enrolment	30.1	36.9	21.3
HIV infection	Negative	Positive	Negative
Pregnancy outcome	Lost to follow-up	Live newborn	Live newborn
Gestational age at delivery	Lost to follow-up	37.3	41.1
Small-for-gestational-age newborn	Lost to follow-up	No	No

*DENV* dengue fever virus, *ZIKV* Zika virus, *PRNT* plaque reduction neutralization assay

Among the 18 participants who were DENV PRNT positive, four tested positive at two different study visits. Thirteen (72.2%) of these 18 participants were DENV confirmed during the follow-up period while five (27.8%) participants were confirmed DENV positive at enrolment. Four (22.2%) of the DENV-positive participants delivered small-for-gestational-age offspring and one (5.6%) participant delivered a newborn with microcephaly (the participant was DENV PRNT positive at enrolment at 11.2 weeks of gestation). Seven (31.8%) of the participants with confirmed DENV were HIV infected and one participant had a stillbirth at 31.3 weeks of gestation.

We found the sera of one mother and her newborn collected during delivery to be seropositive for anti-ZIKV IgM and DENV PRNT. Another mother had maternal serum collected at delivery that was anti-ZIKV IgM positive but ZIKV PRNT negative (therefore not evaluated for DENV) and newborn serum that was anti-ZIKV IgM and DENV PRNT positive.

## Discussion

In this large longitudinal study of pregnant women in coastal Kenya from 2017 to 2019, we found little evidence of active Zika virus circulation, but possible evidence of a higher prevalence of severe microcephaly of 0.2% compared with 0.13% expected from a reference population based on INTERGROWTH-21st standards [18]. However, the prevalence of any microcephaly in our study was less than half of the expected (2.3%) in the reference population. We found evidence of ZIKV neutralizing antibodies in only three among 2293 participants screened first for IgM. Two of the participants were already seropositive for ZIKV by IgM and PRNT at enrolment between 12 and 14 weeks of gestation, and therefore, it remains unclear if the infection occurred during pregnancy or previously. However, of note, 136 of 166 women became IgM positive during follow-up of which 18 were determined positive for DENV by PRNT. With the assumption that the IgM and ZIKV PRNT positivity reflected cross-reaction of dengue IgM antibodies, these results suggest substantial dengue circulation in this population, but since only the ZIKV PRNT positive were tested for DENV, it remains likely that the true burden of dengue was higher. The higher DENV seropositivity reflects high transmission of DENV in an area that has reported regular outbreaks over the last decade [25, 26]. Almost three-quarters of the participants with evidence of exposure to DENV seroconverted during the study follow-up, further evidence of the high level of transmission of DENV.

The prevalence of microcephaly of 90 cases per 10,000 births in our study was about half that reported in a retrospective study (200 cases per 10,000 births) from an earlier period (2012–2016) in Kilifi, coastal Kenya [27]. However, the level of microcephaly reported in our study was at least 9 times higher than < 10 cases per 10,000 births reported from studies in Europe and Latin America [28, 29]. It was also 1.5 times higher than reported in Brazil (60 cases per 10,000 births) at the peak of the ZIKV outbreak in 2016. However, a study in China reported a higher prevalence of 410 cases per 10,000 births [30]. None of the newborns with microcephaly in our study had evidence of ZIKV and only one had confirmed DENV exposure. Nonetheless, the low number of infections in our study means that we cannot exclude an association. Microcephaly is associated with infectious (such as rubella, cytomegalovirus, herpes simplex virus, immunodeficiency virus, toxoplasmosis, and syphilis), environmental, and genetic causes. The higher prevalence of microcephaly in coastal Kenya as reported in our study and from the study in Kilifi [27] compared to facility-based studies from other countries could be because the cited studies used clinical data, in addition, to head circumference measurements to define microcephaly and variations in the head circumference cutoff for defining microcephaly (some studies defined microcephaly head circumference < 3 SD below the mean for sex and gestational age).

The low levels of ZIKV seropositivity in our study are similar to findings from several studies in Africa and Asia. A study in a long-term cohort on HIV transmission among women in Mombasa assessed by PRNT for anti-ZIKV neutralizing antibodies among febrile presentations found ZIKV seropositivity in one of 900 participants [31]. Similar findings with fewer than five participants seropositive for ZIKV from among several hundred tested were reported in Uganda, DR Congo, Cambodia, and Vietnam [21, 32–34]. Commonly in these studies, DENV seropositivity was higher than that of ZIKV. Contrary to our findings, a study in northern Kenya reported a high ZIKV seroprevalence of 7% and DENV seroprevalence of 1%, although it was not clear how the potential cross-reactivity was assessed [12].

The low levels of ZIKV transmission could be due to the competition of the virus on the same vector and host. In countries that reported high ZIKV seroprevalence of 50–73% during the 2013–2016 outbreak, the incidence of DENV decreased during the ZIKV outbreak but increased after the outbreak [35]. Additionally, the differences in transmission could be a reflection of the cyclic variation of flavivirus transmission or other favorable climatic conditions [36]. Recently, it has been shown that different subspecies of *Aedes aegypti* may have different susceptibility to ZIKV infection and that the vectors in the Americas represent a population of mosquitoes susceptible to ZIKV infection, whereas similar mosquitoes in Africa may be resistant [37].

Our findings of IgM positivity in neonatal sera suggest prepartum infection and vertical transmission of DENV since IgM antibodies do not cross the placenta. Vertical transmission of DENV is associated with preterm deliveries and low birth weight although the mechanism is not clear. Congenital malformations of the nervous system have also been associated with DENV infection in pregnancy [38–40].

Our study had several limitations. First, the screening antibody test was ELISA for ZIKA IgM and we did not undertake DENV IgM testing. We may have underestimated the true ZIKV seroprevalence because we only included ZIKV-positive sera from those that were positive by IgM and PRNT and without high titers of DENV antibodies. Some of the sera initially positive for ZIKV by PRNT and then judged as dengue infections because of high DENV titer may have been acute ZIKV infections but with high DENV background neutralizing antibodies due to historic DENV infections. Second, since we were not able to detect any ZIKV virus, we were unable to determine if any circulating ZIKV was from the African lineage or the Asian lineage that spread throughout the Americas and was associated with microcephaly. Third, we did not test systematically for anti-ZIKV IgG which would have indicated true seroprevalence in the cohort and the pre-existing immunity to ZIKV infection. However, the low yield from the ZIKV PRNT overall testing suggests that the background ZIKV seroprevalence is low. Fourth, our assessment of adverse neonatal outcomes was only conducted during delivery. Some of the malformations associated with congenital Zika syndrome become apparent during the months after delivery which our study could not establish because of a lack of infant follow-up. Finally, about one-third of the deliveries were not within the study facilities and we, therefore, could not assess some of the potential adverse outcomes among the newborns. In addition, almost two-thirds of the stillbirths were not examined for congenital abnormalities because the deliveries were not within the study facility, or the bodies were disposed of before the study staff could examine them.

## Conclusions

The epidemiology and transmission of ZIKV in Africa remain unclear. However, we found no evidence of substantial ZIKV transmission in coastal Kenya, and most of the population of coastal Kenya, including pregnant women, are susceptible to infection. Our study highlights the need for enhanced surveillance of arboviral infections and congenital abnormalities in Kenya and throughout Africa. Understanding transmission, lineages of ZIKV, and vector presence and susceptibility is key to risk assessment and future prevention and control of outbreaks.

## Supplementary Information

**Additional file 1:** **Table S1.** Comparison of some characteristics between participants who completed follow-up and thoselost to follow-up.

## Data Availability

The datasets used and/or analyzed during the current study are available from the corresponding author on reasonable request.

## Abbreviations

ANC: Antenatal care
CDC: US Centres for Disease Control and Prevention
DENV: Dengue virus
ELISA: Enzyme-linked immunosorbent assay
HC: Head circumference
IgM: Immunoglobulin M
IQR: Interquartile range
PRNT: Plaque reduction neutralization assay
rRT-PCR: Real-time reverse transcription polymerase chain reaction
SD: Standard deviation
SGA: Small-for-gestational-age
sSA: Sub-Saharan Africa
ZIKV: Zika virus
