# Supplementary material for: Prevalence of microcephaly and Zika virus infection in a pregnancy cohort in Kenya, 2017–2019
Source: BMC Med. 2022 Sep 14;20:291. doi: 10.1186/s12916-022-02498-8 (PMC9470235; doi:10.1186/s12916-022-02498-8)
Supplement: Supplementary file 1 — Additional file 1: Table S1. Comparison of some characteristics between participants who completed follow-up and thoselost to follow-up. [file 12916_2022_2498_MOESM1_ESM.docx]

**Prevalence of microcephaly and Zika virus infection in a pregnancy cohort in Kenya, 2017-2019**

Additional File 1: Table S1 Comparison of some characteristics between participants who completed follow-up and those lost to follow-up

| **Characteristic** | **Total** | **Completed Follow-Up** | |  |  |
| --- | --- | --- | --- | --- | --- |
|  |  | **Yes** | **No** | **P-value** |  |
| Age at enrolment in years, median (IQR) | 2312 | 26.7 [22.7-30.1] | 28.3 [24.4-32.5] | <0.001* |  |
| Gestational age in weeks at enrolment, median (IQR) | 2312 | 19.1 [15.4-23.1] | 20.4 [16.0-24.1] | 0.028* |  |
| Education level completed | 2278 |  |  |  |  |
| Primary and below n (%) |  | 490 (26.0) | 102 (26.0) | 0.098 |  |
| Secondary n (%) |  | 857 (45.5) | 159 (40.5) |  |  |
| Tertiary n (%) |  | 538 (28.5) | 132 (33.6) |  |  |
| History of Chronic Disease^a^ n (%) | 2312 | 117 (6.1) | 26 (6.6) | 0.819 |  |
| *Kruskal-Wallis test used as a test of statistical significance IQR – Interquartile range ^a^Chronic disease included Asthma, hypertension, diabetes and epilepsy | | | | | |
